# Approach to the Adolescent With Substance Use in the Acute Setting

**DOI:** 10.7759/cureus.16309

**Published:** 2021-07-11

**Authors:** Sujetha Sathanantham, Kavinda Dayasiri, Vijayakumary Thadchanamoorthy

**Affiliations:** 1 Preliminary Care Unit, Lady Ridgeway Hospital, Colombo, LKA; 2 Paediatrics, Faculty of Medicine, University of Kelaniya, Ragama, LKA; 3 Clinical Sciences, Faculty of Health Care Sciences, Eastern University, Batticaloa, LKA

**Keywords:** substance, adolescent, intoxication, supportive care, mental health

## Abstract

Psychoactive substance use during adolescence is an emerging challenge to the public health system due to the potential negative impact on the emotional, cognitive, social, physical and academic outcomes of adolescents. An increase in autonomy during adolescence, peer influence and willingness to experiment, lead to taking high-risk decisions subsequently prompting towards substance abuse and alcohol use. Substance use is heterogeneous among adolescents, which differs with availability, perceptions of use and specific drug usage. In adolescents’ substance abuse, beyond emergency care, brief counselling with psychosocial assessment and follow up are recommended for successful management.

The article reviews the common substances used by adolescents, types of presentations, clinical evaluation of patients and their background, laboratory testing, emergency management and follow up for long-term management.

## Introduction and background

Adolescence is defined by the World Health Organization (WHO) as “the transition period from childhood to adulthood ranging from ages 10 to 20 years.” Adolescents contribute to 16.1% of the total population of Sri Lanka and approximately, 70% of them attend school [1]. According to a survey conducted in Sri Lanka in 2016, 2.7% of schooling students consume addictive substances [1]. Adolescence is a most challenging developmental period, especially consisting of biological, psychological, social and cognitive development. Further, peer pressure and greater autonomy from parents contribute to risk-taking behaviour and exploration, which are major factors for adolescents’ substance abuse [2]. The commonly consumed substances during this period are cannabis, stimulants, opioids and hallucinogens. Moreover, adolescents have a high risk of experiencing several negative consequences, such as non-fatal and fatal injuries, academic failure, violence, addiction, suicidal attempts, sexually transmitted infections and unintended pregnancy, due to substance consumption [2].

While encountering adolescents with suspicion for substance abuse in an emergency setting, physicians have to elicit detailed history and physical examination to discover specific toxidromes such as anxiety with intractable nausea and vomiting in cannabis intoxication and respiratory depression and miosis in opioid intoxication. However, the manifestations of substance use extend beyond specific toxidromes mainly due to poly-drug usage [3]. A timely and efficient therapeutic approach is essential in an emergency setting because rapid clinical deterioration can lead to life-threatening complications.

## Review

Clinical presentation

Adolescents have limited insight regarding the threats of substance consumption. Acute unpleasant symptoms or medical complications following consumption lead to emergency care visits. However, some people present with suicidal attempts, mental health disorders such as anxiety disorders, psychotic disorders, and mood disorders, and participate in risky behaviour such as driving under the influence of substances or engaging in sexual activities [3].

Evaluation in an acute setting

Adolescents with substance abuse are usually brought to the emergency department by concerned family members. Obtaining collateral information from family members or other sources will assist in clinical evaluation. Maintaining a non-judgmental, open-minded approach towards adolescents and accompanying family members facilitates the acquisition of accurate information and helps to better understand adolescent’s circumstances. Careful history taking and focused examination help in risk assessment and management (Table 1) [3].

**Table 1:** Initial assessment of suspected substance overdose

History	Age and Gender
	Warning features
	Drug exposure history (timing, drug(s), dose, route, type of ingestion (recreational, deliberated self-poisoning, accidental)
	Past drug history and treatment (alcohol, tobacco)
	Impact of substance use on the patient’s behavioural, psychological, cognitive, and physiological functioning
	Medical history
	Psychiatric history and treatment
Physical examination	General appearance: agitation, coma, impaired consciousness
	Vital signs: respiratory rate and effort, saturation, heart rate, blood pressure, temperature
	Observations: colour, smell, skin changes, needle marks
	Neurological: GCS, pupil, limb movement, tone, fasciculation, reflexes, clonus
	Others: signs of trauma
Investigations	Full blood count
	Blood sugar
	Blood biochemistry (electrolytes, renal profile)
	Blood gas
	Electrocardiogram, Troponin
	Creatine kinase
	Specific blood tests: alcohol level, paracetamol level and salicylate levels
	Urine / saliva and hair test for illicit drugs

The managing clinician should be aware that adolescents are a high-risk population for multiple substance ingestion. Therefore, it is essential to gather comprehensive history, including details of substance use such as alcohol, caffeine, tobacco, over-the-counter medications, herbal preparations and prescription medication. The history of pre-existing medical conditions is important for the effective management and excluding other organic pathologies. Mental health assessment is crucial to differentiate pre-existing psychiatric illness or mental illness linked with substances and to assist subsequent psychiatric referral [3,4].

Illicit substance users often use multiple terminologies. Table 2 contains common street names for substances and the routes of administration that may aid conversation with patients [4,5].

**Table 2:** Common street names for substances and routes of administration MDMA (3,4-Methyl​enedioxy​methamphetamine), PMA (Paramethoxyamphetamine)

Drug name	Street name	Route	Duration of effect
Cannabis	Marijuana, Ganja, Weed, Pot, Grass, Hash, Joint, Mull, Dope, Cone	Smoked, oral	1–3 h
Amphetamines (methamphetamine, PMA)	Speed, Meth, Ice, Jib crystal, Uppers, Tina	Oral, snorted, smoked, injected (IM)	6–8 h if taken orally or injected, 10–12 h if smoked
Cocaine	Snow, Crack, Coke, Rock, C, Flake, Candy, Charlie	Snorted, injected (IV), smoked (crack)	15–30 min if snorted 5–10 min if smoked
Ecstasy (MDMA)	XTC Eccies, M&Ms, E, X, Adam, Hug drug	Oral, snorted	4–6 h (dependent on dose)
Heroin	Smack, Horse, Junk, Ska, Brown, Harry, “H”, Speedball (injected with cocaine)	Smoked, injected (SC, IM, IV), snorted, oral (pills)	3–5 h
γ-hydroxybutyrate (GHB)	Fantasy, Liquid G, Date rape drug, Gina, Grievous bodily harm, Cherry meth, Liquid Fanta, Easy Lay, Georgia Homeboy, Liquid Ecstasy	Oral, snorted	1.5–3 h, longer with alcohol
d-lysergic acid diethylamide (LSD)	Acid, Trips (high form), Acid Blotter, Dots, Boomers, Yellow sunshine	Oral	8–12 h
Ketamine	K, Special K, Super K, Ketalar, Green	Intranasal, by injection, oral	1–2 h
Benzodiazepines	Candy, Tranks, Downers, Sleeping pills, Xanax, Ativan, Valium	Oral	Last up to 24 h if long-acting

Adolescents are more vulnerable to use multiple substances to achieve their “high” compared to adults. Recognition of specific group/ toxidromes will facilitate the management, even when the specific substance is unknown. Table 3 summarises the physical finding of different substances [4,6].

**Table 3:** Acute signs and symptoms of substance use in adolescents MDMA (3,4-Methyl​enedioxy​methamphetamine)

	Respiratory	Cardiovascular/Autonomic	Central Nervous System (CNS)	Gastrointestinal
Cannabis	Frequent respiratory infections, cough	Tachycardia, sweating, tremors, high fever, chills	Psychological disturbance: depersonalisation, decreased inhibition, disorientation, altered mood, lack of attention, memory impairment	Abdominal pain, hyperemesis syndrome
Opioids (heroin, morphine, codeine, oxycodone, and fentanyl)	Respiratory depression, pulmonary oedema	Autonomic effects: miosis sweating, hypothermia Cardiovascular effects: bradycardia, hypotension	CNS depression: drowsiness to coma	Dry mouth, nausea, vomiting, constipation
Stimulants (cocaine, amphetamine and MDMA)	Pulmonary oedema, respiratory distress due to pulmonary barotrauma	Autonomic effects: mydriasis, hyperthermia, flushing, diaphoresis Cardiovascular effects: hypertension, tachycardia, arrhythmias myocardial depression	CNS excitation: agitation, euphoria, delirium, hallucinations, psychosis, seizures neuromuscular excitation: hyper-reflexia, tremor	Nausea, stomach pain
Benzodiazepines (lorazepam, alprazolam, diazepam, clonazepam)	Respiratory depression	Hypotension, bradycardia	CNS depression, slurred speech, ataxia, drowsiness, confusion, impaired judgment	
Gamma hydroxybutyrate (GHB)	Rapid onset of respiratory depression	Miosis, hypothermia, bradycardia	CNS depression, anterograde amnesia, hallucinations, euphoria, disinhibition, sociability	Nausea, vomiting, abdominal pain
LSD (d-lysergic acid diethylamide)		Autonomic effects: mydriasis, hyperthermia, sweating cardiovascular effects: tachycardia, hypertension,	Delusion, hallucinations, impaired motor coordination, perceptual distortion, agitation	Nausea, loss of appetite
Ketamine	Difficulty in breathing	Tachycardia, hypertension	Impaired consciousness, euphoria, altered perceptions, mystical experiences	Abdominal pain

Differential diagnosis

There are a number of differential diagnoses for altered mental status or agitation, which need to be actively ruled out in those who present with suspicious substance intoxication. Hypoglycaemia is an easily reversible differential diagnosis. Meningitis and encephalitis need to be considered in those who present with a history of fever, headache, vomiting followed by altered mental status and nuchal rigidity. Sepsis has to be considered in systemically ill adolescents with foci of infection [7,8]. Headache, recent onset behavioural changes, focal neurological deficits and seizure can be the presentation of a space-occupying lesion.

Overdose of over-the-counter medications leading to anticholinergic toxidrome, salicylate overdose, and serotonin syndrome are also important differential diagnoses in adolescents with deliberate self-harm and substance use. Recent history of trauma/accidents warrants ruling out head injury. Heat exhaustion/heat stroke is another differential diagnosis that correlates with environmental temperature and outdoor prolonged sports/exercise [8]. Thyrotoxicosis and pheochromocytoma have to be considered in adolescents with suggestive systemic clinical features such as eye signs, paroxysmal headache, sweating and palpitation; however, they are uncommon. Psychosis/schizophrenia can be a diagnosis of exclusion and correlates with background and family history of psychiatric illness [9].

Investigations

Acute intoxication features may overlap with sepsis or encephalitis, which may warrant sepsis work up such as full blood count, C-reactive protein (CRP) and cultures based on the focus of infection. Blood sugar should be determined to exclude hypoglycaemia. Urine human chorionic gonadotropin (HCG) should be done in all adolescent females to exclude unintended pregnancy. Blood gas assessment is helpful in identifying metabolic and respiratory acidosis and oxygenation. Creatine kinase is performed in patients with delirium or aggressive behaviour, hyperthermia and seizures to identify rhabdomyolysis. In substance abuse, electrocardiography (ECG) is essential in the detection of acute coronary syndrome and arrhythmias. It also helps to identify various changes due to electrolyte disturbances. A chest x-ray may be indicated in those with pulmonary barotrauma (spasmodic coughing following smoke inhalation), pneumonia, or pulmonary oedema [3,8].

Blood alcohol level, blood paracetamol and salicylate levels are warranted in suspected co-ingestions. Drug testing has limited value in the diagnosis of substance abuse and they often do not quantify the severity of ingestions. Further, a negative drug value does not exclude the presence of substance use, especially with newer substances [3].

Management in the acute setting

Intoxicated patients should be assessed through the ABCDE approach. Early resuscitation is a life-saving measure in intoxicated patients. The management of substance overdose primarily involves supportive care [10,11].

Airway and antidotes: CNS depression, altered level of consciousness, and hypersecretion lead to a threatened airway. Ensure the airway patency, which may necessitate intubation. Consider emergency empiric administration of antidotes following the establishment of the secured airway.

Breathing: Facilitate breathing with supplemental oxygen. Monitor oxygen saturation during management. Ventilator support is needed in lethal intoxication.

Circulation: Initiate cardiac monitoring and electrocardiographic studies. Intravenous access should be secured to ensure fluid administration. Adequate hydration is essential in intoxication.

Disability: Manage the patient in a calm environment. Verbal de-escalation is an important initial management step of agitation. If a poor response or aggressive behaviour, pharmacological management should be considered including the use of benzodiazepines. Exclude hypoglycaemia, which is a rapidly reversible cause of the agitation. Seizures should be actively managed with benzodiazepine [12]. Treat cardiac dysrhythmias and metabolic abnormalities.

Exposure: Manage hyperthermia by effectively cooling measures. Treat concomitant injuries.

Following initial stabilization, it is important to obtain a rapid history and thorough physical examination to look for an underlying toxic syndrome. This assessment will help in the early administration of decontamination and specific treatment.

Management of specific substances

Cannabis

The main clinical manifestations of cannabis intoxication are panic attacks, anxiety and autonomic instability. Acute toxicity is rarely serious and the main-stay of management is reassurance and supportive management. Benzodiazepines may be helpful to control agitation [3,9].

Opioids

The main clinical features of opiate overdose include respiratory depression, miosis, bradycardia and stupor or coma. They may present with complications such as pulmonary oedema and aspiration pneumonitis. Determination of PCO2 level by blood gases is an assessment modality for hypoventilation. In mild to moderate cases, specific treatment is usually not necessary. The priority of management is the protection of the airway and giving ventilatory support. Naloxone, an opioid antagonist, is the antidote for opioid intoxication and should be titrated according to respiratory effort and rate. If long-acting opiate has been ingested, the patient may need IV naloxone infusion (rate set as 60% of initial bolus) as well as the prolonged observation [6,9]. The half-life of naloxone is shorter than opioids and this may lead to rebound opioid toxicity. Therefore, cardio-respiratory monitoring is crucial following initial stabilisation.

Stimulants: Amphetamines, Cocaine and MDMA

Sympathomimetic excess is characterized by autonomic and cardiovascular hyper-stimulation with neuropsychiatric clinical features such as agitation, delirium and hallucinations. The primary focus is calming the patient to prevent harm to the patient as well as to staff. Therefore, they have to be managed in a calm and low stimulant environment. Monitoring of vital signs including respiratory rate, heart rate, blood pressure and temperature is crucial in management. Benzodiazepines are the first-line medication for agitation or aggressive behaviour. Neuroleptics can be used if delusions or hallucinations are present. Hyperthermia can be fatal and should be treated actively. Institution of aggressive cooling measures and reduction of activity with benzodiazepines (even consider paralysis) are treatment measures for hyperthermia [7,8].

Severe hypertension can cause cardiovascular and cerebrovascular complications. High blood pressure should be controlled with rapidly acting antihypertensive medications such as sodium nitroprusside (vasodilator) and phentolamine (alpha-adrenergic blocker) [8,13]. Chest pain warrants assessment for possible myocardial ischemia or infarction. The stimulant-induced acute coronary syndrome should be managed with nitrates, benzodiazepines, opiates and aspirin initially. If ischemia persists, it warrants standard therapy. Wide-complex tachyarrhythmias can be associated with cocaine overdose due to sodium channel blockade, and sodium bicarbonate is the treatment option [13].

Benzodiazepines

Respiratory depression and mental depression are the main clinical features of benzodiazepine overdose. The mainstay of management for acute intoxication is supportive care. Monitoring of vital signs including respiratory rate, oxygen saturation, level of consciousness and blood gas is essential in management. Acute management consists of maintaining the airway, facilitating ventilation with oxygenation and haemodynamic support. Flumazenil, a benzodiazepine antagonist, can be used in severe intoxication associated with respiratory and neurological depression [14]. If there is no reversal within 5-10 minutes of use of flumazenil, other diagnoses should be considered.

Other Substances

Consumption of newer drugs, such as gamma hydroxybutyrate (GHB), Ketamine and lysergic acid diethylamide (LSD), is mainly observed in club environments. Most of the drugs are consumed with other substances, especially alcohol. Mostly, new substance consumptions are misdiagnosed due to non-specific features and clinical features similar to other toxidromes including GHB intoxication, and intoxication of benzodiazepines, and barbiturates [15].

GHB is a short-acting clear liquid that causes CNS depression. The clinical features are similar to sedative-hypnotic drugs overdose. Management is mainly supportive care; intubation might be needed to maintain airway patency and protect from aspiration [16]. LSD is a prominent hallucinogenic drug. Management of LSD intoxication is mainly supportive. Agitation and psychosis associated with LSD intoxication may need benzodiazepines [17].

Complications

Acute intoxication or repeated use of substances can cause multiple complications. Acute intoxication causes damage to multiple systems including the cardiovascular, respiratory, cerebrovascular and renal systems. Complications mainly depend on the type of substance, ingested dose, route of administration, and associated comorbidities and risky behaviour under the influence of the substance. Stimulant abuse leads to cardiovascular and cerebrovascular complications due to catecholamine excess. In the cardiovascular system, it causes arrhythmias, myocardial ischemia or infarction and cardiomyopathy. Especially cocaine is linked with wide complex tachyarrhythmias due to sodium channel blockade [7]. Cerebrovascular complications such as seizures, ischemic strokes and subarachnoid or intracerebral haemorrhages can occur due to direct cerebrovascular involvement [8].

Respiratory complications due to substance intoxication include pulmonary haemorrhage, pneumonia, pulmonary oedema, pneumothorax, pneumomediastinum, aspiration pneumonitis and respiratory arrest [11]. Further, bronchospasm is another frequent clinical presentation in smokers. Pulmonary barotrauma may be a consequence of spasmodic coughing with a sudden increase in airway pressure following smoke inhalation [13]. “Crack lung” is an acute pulmonary syndrome associated with smoking crack cocaine and leads to diffuse alveolar damage and haemorrhagic alveolitis within 48h of smoking [18].

Delirium, seizure and hyperthermia can cause rhabdomyolysis leading to acute kidney injury. Infections are another entity of complications that arise due to parenteral use of substances or disinhibition causing participation in high risk or unprotected sexual activity. Parenteral use of substances causes blood-borne transmission of hepatitis B, C, and D and HIV/AIDS. Intravenous cocaine users are at high risk of infective endocarditis than other parenteral drug users [13]. Failure to intervene at an early stage to prevent substance consumption in adolescence often leads to substance addiction and dependence.

Follow up beyond the emergency setting

Adolescents with substance abuse should be collaboratively managed by primary care physicians and psychiatrists, beyond emergency care. Effective management should address holistic management of the patients and not just their substance use. Most of these adolescents have underlying psychiatric conditions, which have to be considered in their management [9]. Therefore, proper psychiatric care is essential for successful long-term management. Depending on the severity of the substance abuse disorder, treatment and follow up should be arranged at the appropriate level of care. This may include mental health counselling or specialized treatment programs in inpatient drug rehabilitation facilities [19].

## Conclusions

Adolescent substance use is an emerging burden on the healthcare system. Effective management of acute substance intoxication requires accurate risk assessment, understanding of the pharmacological properties of the substance, and appropriate supportive care. Education of the adolescent patients and their family members on the immediate effects and long-term consequences of substance use including psychosocial and medical impact is essential in management. A collaborative team approach with family, primary care physicians and mental health providers is a key factor in the effective management of adolescents with substance abuse.
